# The impact of hypoglycaemia on daily functioning among adults with diabetes: a prospective observational study using the Hypo-METRICS app

**DOI:** 10.1007/s00125-024-06233-1

**Published:** 2024-07-30

**Authors:** Uffe Søholm, Melanie Broadley, Natalie Zaremba, Patrick Divilly, Petra Martina Baumann, Zeinab Mahmoudi, Gilberte Martine-Edith, Julia K. Mader, Monika Cigler, Julie Maria Bøggild Brøsen, Allan Vaag, Simon Heller, Ulrik Pedersen-Bjergaard, Rory J. McCrimmon, Eric Renard, Mark Evans, Bastiaan de Galan, Evertine Abbink, Stephanie A. Amiel, Christel Hendrieckx, Jane Speight, Pratik Choudhary, Frans Pouwer, on behalf of the Hypo-RESOLVE consortium

**Affiliations:** 1 Medical & Science, Patient Focused Drug Development, Novo Nordisk A/S, Søborg, Denmark; 2 Department of Psychology, University of Southern Denmark, Odense, Denmark; 3 Department of Diabetes, School of Cardiovascular and Metabolic Medicine and Sciences, Faculty of Life Sciences and Medicine, King’s College London, London, UK; 4 Division of Endocrinology & Diabetology, Medical University of Graz, Graz, Austria; 5 Data Science, Department of Pharmacometrics, Novo Nordisk A/S, Søborg, Denmark; 6 Division of Endocrinology and Diabetology, Department of Internal Medicine, Medical University of Graz, Graz, Austria; 7 Department of Endocrinology and Nephrology, Nordsjællands Hospital Hillerød, Hillerød, Denmark; 8 Steno Diabetes Center Copenhagen, Herlev, Denmark; 9 Lund University Diabetes Center, Lund University, Malmö, Sweden; 10 Department of Endocrinology, Skåne University Hospital, Malmö, Sweden; 11 Department of Oncology and Metabolism, University of Sheffield, Sheffield, UK; 12 Institute of Clinical Medicine, University of Copenhagen, Copenhagen, Denmark; 13 Systems Medicine, School of Medicine, University of Dundee, Dundee, UK; 14 Department of Endocrinology, Diabetes, Nutrition, Montpellier University Hospital, Montpellier, France; 15 Institute of Functional Genomics, University of Montpellier, CNRS, Inserm, Montpellier, France; 16 Welcome MRC Institute of Metabolic Science and Department of Medicine, University of Cambridge, Cambridge, UK; 17 Department of Internal Medicine, Radboud University Medical Centre, Nijmegen, the Netherlands; 18 Department of Internal Medicine, Division of Endocrinology and Metabolic Disease, Maastricht University Medical Centre, Maastricht, the Netherlands; 19 CARIM School for Cardiovascular Diseases, Maastricht University, Maastricht, the Netherlands; 20 Institute for Health Transformation, School of Psychology, Deakin University, Geelong, VIC Australia; 21 The Australian Centre for Behavioural Research in Diabetes, Diabetes Victoria, Carlton, VIC Australia; 22 Diabetes Research Centre, University of Leicester, Leicester, UK; 23 Steno Diabetes Center Odense (SDCO), Odense, Denmark

**Keywords:** Daily functioning, Ecological momentary assessment, Hypoglycaemia, Quality of life

## Abstract

**Aims/hypothesis:**

The aim of this work was to examine the impact of hypoglycaemia on daily functioning among adults with type 1 diabetes or insulin-treated type 2 diabetes, using the novel Hypo-METRICS app.

**Methods:**

For 70 consecutive days, 594 adults (type 1 diabetes, *n*=274; type 2 diabetes, *n*=320) completed brief morning and evening Hypo-METRICS ‘check-ins’ about their experienced hypoglycaemia and daily functioning. Participants wore a blinded glucose sensor (i.e. data unavailable to the participants) for the study duration. Days and nights with or without person-reported hypoglycaemia (PRH) and/or sensor-detected hypoglycaemia (SDH) were compared using multilevel regression models.

**Results:**

Participants submitted a mean ± SD of 86.3±12.5% morning and 90.8±10.7% evening check-ins. For both types of diabetes, SDH alone had no significant associations with the changes in daily functioning scores. However, daytime and night-time PRH (with or without SDH) were significantly associated with worsening of energy levels, mood, cognitive functioning, negative affect and fear of hypoglycaemia later that day or while asleep. In addition, night-time PRH (with or without SDH) was significantly associated with worsening of sleep quality (type 1 and type 2 diabetes) and memory (type 2 diabetes). Further, daytime PRH (with or without SDH), was associated with worsening of fear of hyperglycaemia while asleep (type 1 diabetes), memory (type 1 and type 2 diabetes) and social functioning (type 2 diabetes).

**Conclusions/interpretation:**

This prospective, real-world study reveals impact on several domains of daily functioning following PRH but not following SDH alone. These data suggest that the observed negative impact is mainly driven by subjective awareness of hypoglycaemia (i.e. PRH), through either symptoms or sensor alerts/readings and/or the need to take action to prevent or treat episodes.

**Graphical Abstract:**

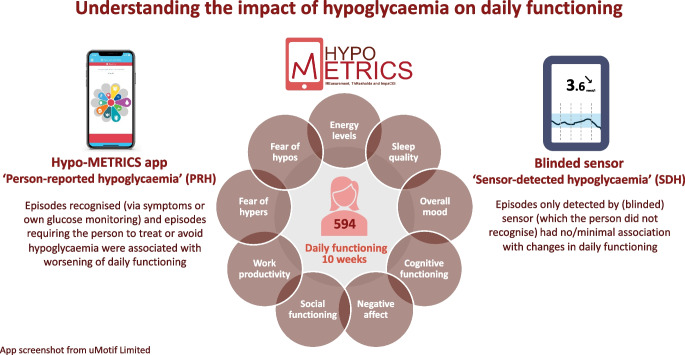

**Supplementary Information:**

The online version of this article (10.1007/s00125-024-06233-1) contains peer-reviewed but unedited supplementary material.


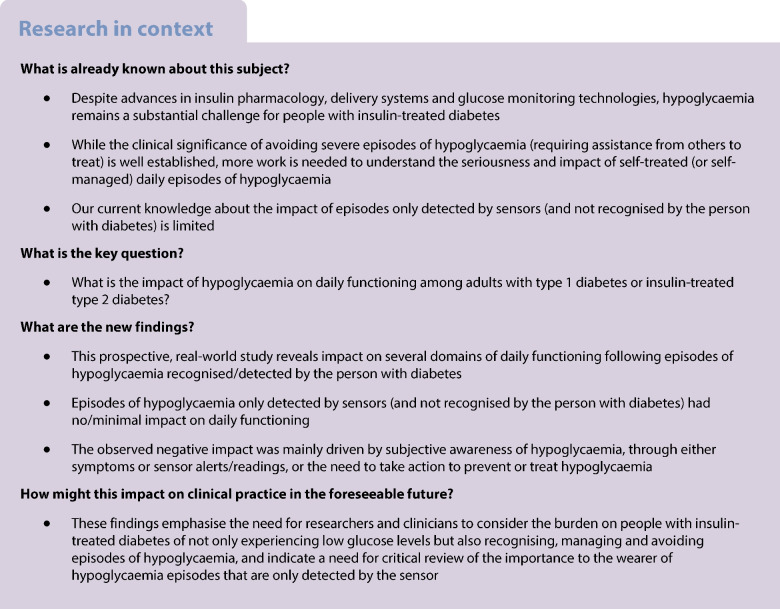


## Introduction

Despite advances in insulin pharmacology, delivery systems and glucose monitoring technologies, hypoglycaemia remains a substantial challenge for people with insulin-treated diabetes. Adults with type 1 diabetes experience approximately two self-treated episodes per week [1]. Although fewer episodes are experienced by adults with insulin-treated type 2 diabetes, the frequency increases over time along with more individuals transitioning to insulin treatment [1]. Hypoglycaemia can occur unexpectedly and can lead to dangerous situations such as cognitive impairment, coma and, rarely, death [2]. Further, hypoglycaemia has a negative impact on multiple aspects of quality of life (QoL) [3, 4].

Person-reported hypoglycaemia (PRH, sometimes referred to as self-reported hypoglycaemia) has earlier been defined as episodes that the person reports due to the experience of symptoms or having knowledge of a low glucose level from a measurement or alarm [5]. Sensor-detected hypoglycaemia (SDH) has been defined by consensus as episodes of hypoglycaemia captured via continuous glucose monitoring (CGM) and lasting at least 15 min below a given threshold (either 3.9 mmol/l or 3.0 mmol/l) [6]. As previously shown, PRH is prone to under-reporting compared with SDH [4], which may be partly due to inconsistency in definitions [4]. Recent findings have highlighted that some episodes of PRH are associated with SDH (PRH and SDH episode), while others are only perceived by the person but not confirmed by the sensor (PRH only) and yet others are only detected by the sensor without being recognised by the individual (SDH only) [7]. There is an urgent need for assessment of whether PRH and/or SDH impact daily life of people with diabetes. In particular, our current knowledge about the impact of episodes only detected by sensors (and not recognised by the person with diabetes) is limited. While the clinical significance of avoiding severe episodes of hypoglycaemia (requiring assistance from others to treat because of cognitive dysfunction) has been well established [4, 8–10], more work is needed to understand the seriousness and impact of self-treated (or self-managed) episodes of hypoglycaemia.

Validated measures used for assessing the impact of hypoglycaemia (e.g. on wellbeing or QoL [4]) typically assess this across several days, weeks or months after episodes occur. This may result in recall bias regarding the frequency and/or severity of episodes and a loss of granularity about their temporal impact [11]. Recent advances in technologies enable the opportunity to link experiences with actual glucose levels. Although some studies have prospectively explored the daily impact of hypoglycaemia, it has been suggested that multiple daily assessments may be necessary to assess outcomes temporally closer to hypoglycaemic episodes [12]. However, existing measures have not been specifically designed or validated to capture the daily impact of hypoglycaemia. The Hypo-METRICS (Hypoglycaemia MEasurement, ThResholds and ImpaCtS) smartphone app was developed for the purpose of capturing the impact of hypoglycaemia on daily functioning (such as sleep quality, mood, energy levels and other domains that might be impacted by hypoglycaemia, and that can vary from day to day), in a close-to-real-time manner [13, 14].

The aim of this study was to determine the impact of hypoglycaemia among adults with type 1 diabetes and insulin-treated type 2 diabetes on daily functioning. First, we tested the hypothesis that daily functioning will differ significantly on days or nights with and without PRH and/or SDH. Second, we exploratively assessed the impact of PRH subtypes (e.g. whether the individual reported their episode due to symptoms or a glucose measurement) and blinded SDH subtypes (i.e. glucose levels <3.9, <3.0 or ≤2.2 mmol/l) on daily functioning.

## Methods

### Study design

The Hypo-METRICS study is a prospective observational study involving nine clinical centres across five European countries and is part of the EU IMI2 Hypo-RESOLVE (Hypoglycaemia – REdefining SOLutions for better liVEs) programme [15]. Ethical approval was granted in each of the five countries. The protocol has been published [16]. Briefly, the study involved the following elements: (1) assessment of daily functioning, captured with the Hypo-METRICS app [13], three times daily for 70 days; (2) continuous measurement of interstitial glucose via a blinded sensor (Abbott FreeStyle 2 Libre; Alameda, CA, USA) (i.e. data unavailable to the participants) modified to collect data every 5 min; (3) baseline collection by research staff of demographic and diabetes-related information; and (4) completion of validated self-reported outcome measures online (via Qualtrics, Provo, UT) at baseline and 10 weeks of follow-up and via the Hypo-METRICS app (daily and weekly). Participants were eligible if they were aged ≥18 years and belonged to one of the following three groups: (1) type 1 diabetes with intact awareness of hypoglycaemia (Gold score <4 [17]); (2) type 1 diabetes with impaired awareness of hypoglycaemia (Gold score ≥4 [17]); or (3) type 2 diabetes managed with at least one insulin injection per day. Participants also needed to have had at least one episode of hypoglycaemia (symptomatic or confirmed by glucose measurement) in the past 3 months. Participants were recruited via the study sites and via online and offline advertisement. They provided informed consent.

### Ethical considerations

The Hypo-METRICS clinical study has received ethical approval at the lead site from the South Central Oxford B Research Ethics Committee (20/SC/0112) and in the other European countries (Ethikkommission der Medizinischen Universität Graz [Austria], Videnskabsetisk Komite for Region Hovedstaden [Denmark], Comité De Protection Des Personnes SUD Mediterranne IV [France] and Commissie Mensgebonden Onderzoek Regio Arnhem-Nijmegen [the Netherlands]). The ClinTrials.gov registration number is NCT04304963.

### Study measures

#### Assessment of daily functioning: The Hypo-METRICS app

The Hypo-METRICS app assesses PRH and aspects of daily functioning, including sleep quality, energy level, overall mood, negative affect, cognitive functioning, daily memory, productivity, social functioning, fear of hypoglycaemia later that day or while asleep and fear of hyperglycaemia later that day or while asleep [14] (see electronic supplementary material [ESM] Table 1 for wording and scoring of the app domains as well as timing of assessments). Via the app, combinations of 29 unique questions were administered daily at three pre-defined time intervals (‘check-ins’: morning 07:00 hours, afternoon 15:00 hours, evening 21:00 hours). Notifications reminded participants to respond to each of the check-ins. The app has been shown to have satisfactory psychometric properties and content validity with high completion rates [14, 18].

#### PRH and SDH

PRH was captured via the Hypo-METRICS app, where participants were asked (in the morning and evening check-in) to report daytime or night-time symptomatic episodes that resolved on ingestion of carbohydrate or had a glucose reading on their own sensor or glucometer <4.0 mmol/l, as well as episodes that were imminent but prevented (note: prevented episodes were excluded for the purpose of the primary Hypo-METRICS objective but included as PRH for the current analyses). SDH was defined as a glucose reading on the blinded sensor <3.9 mmol/l for ≥15 min [6]. The submission times of each morning and evening check-in were used to divide each 24 h period into night-time and daytime intervals. For each participant, each time interval was categorised by presence (+) or absence (−) of PRH and SDH. Each time interval was classified into one of the four following categories depending on PRH and SDH status:

- Type A: PRH (−) and SDH (−), no hypoglycaemia
- Type B: PRH (−) and SDH (+), SDH only
- Type C: PRH (+) and SDH (−), PRH only
- Type D: PRH (+) and SDH (+), both PRH and SDH

For PRH, participants answered follow-up questions about how the episode was detected (PRH detection) and managed (PRH management). These PRH subtypes were coded via an ordered ranking system (see ESM Fig. 1) and subsequently merged into one combined variable to use in the regression model. For the statistical analyses of the current study, severe episodes of hypoglycaemia (confirmed by two independent healthcare professionals) were excluded. While the study-provided sensor was blinded (i.e. data unavailable to the participants), participants continued with their usual means of glucose monitoring (i.e. their own sensor or finger prick).

For the additional exploratory analyses, each time interval was similarly classified based on PRH, resulting in the following subtypes:

PRH detection variable:

- No PRH: no PRH reported at the check-in
- Symptomatic PRH: episodes detected via symptoms
- Asymptomatic PRH: episodes detected via their own glucose monitoring device

PRH management variable:

- No PRH: no PRH reported at the check-in
- Prevented PRH: episodes considered prevented
- Treated PRH: episodes considered treated
- Other PRH: episodes considered neither prevented nor treated

Finally, following the ATTD consensus guidelines [19], the CGM data from the blinded sensors was used to classify each time interval into the following SDH subtypes:

- No SDH: no SDH captured with the blinded sensor
- SDH_3.9_: glucose levels <3.9 but ≥3.0 mmol/l for at least 15 min
- SDH_3.0_: glucose levels <3.0 but >2.2 mmol/l for at least 15 min
- SDH_2.2_: glucose levels ≤2.2 mmol/l for at least 15 min

If more than 30% of the sensor data in each time interval were missing, the entry was excluded from the analysis [6, 20].

### Statistical analyses

Statistical analyses were conducted using R (version 4.2.1) and Rstudio (version 2023.3.1.446) [21]. Sample size calculations were based on the primary Hypo-METRICS study objective [16]. Participants were excluded from analyses if there were no PRH or SDH entry in the dataset. Participant characteristics are presented as number and percentage or mean ± SD. Due to repeated assessments (multiple check-ins) for each participant, the data exhibit a two-level nested structure. Therefore, a multilevel linear regression analysis, with participant ID as a random effect, was used to test the associations between PRH/SDH status in the night-time and daytime and the raw score on each domain of daily functioning in the morning (referred to as ‘morning functioning’, including ten domains) and evening (referred to as ‘evening functioning’, including nine domains) check-ins respectively. Some domains (i.e. daily memory, productivity and social functioning) were only assessed for the evening check-in but were analysed in terms of their associations to both night-time and daytime PRH/SDH status (and are therefore also included in the morning functioning domains). Afternoon check-in data were not included for the current analyses and only questions with 0–10 response scales were included (i.e. excluding work-specific items, which will be reported in a separate study). Missing data were handled using pairwise deletion. After seven model iterations, the final model consisted of the following elements: (1) a multilevel linear regression model assessed using the robustlmm R package for robust estimation [22] due to suboptimal distributions of residuals (including heteroscedasticity); (2) adjustment for autocorrelation; and (3) consensus-guided (agreed upon by members of the Hypo-RESOLVE consortium) list of control variables comprising baseline demographic, clinical, psychological and app-related factors (full list available in ESM Table 2). A Bonferroni-corrected *p* value <0.0002 (calculated based on ten morning domains plus nine evening domains, two separate analyses for type of diabetes and seven model iterations: 0.05/266=0.0002) was applied for the regression models, except on the secondary, and explorative, analyses on PRH and SDH subtypes. For ease of interpretation, figures show regression coefficients transformed to a percentage change in score (on each domain) from the intercept with 95% CIs and are presented for type 1 diabetes and type 2 diabetes separately.

## Results

### Participant characteristics

Table 1 shows the characteristics of the 594 participants (274 with type 1 diabetes and 320 with type 2 diabetes) with available data for analyses. Mean ± SD age was 44.9±16.0 and 61.9±10.2 years and diabetes duration was 23.8±15.6 and 20.4±8.9 years for participants with type 1 diabetes and type 2 diabetes, respectively. Compared with participants with type 1 diabetes, those with type 2 diabetes were significantly older, were more likely to be male and of Asian or Black ethnicity, and were less likely to be in paid employment or study or have higher education. People with type 2 diabetes spent significantly more time in range (glucose ≥3.9, ≤10mmol/l), less time below range (glucose <3.9mmol/l), a greater percentage using finger prick rather than CGM to monitor glucose levels, and had more depressive symptoms, greater perceived cognitive difficulties, lower fear of hypoglycaemia and higher completion of evening check-ins (Table 1).

**Table 1:** Participants’ demographic, clinical, psychological and app-related characteristics by diabetes type

	Type 1 diabetes (*N*=274)	Type 2 diabetes (*N*=320)	*p* value^a^
Demographics			
Age, years	44.9±16.0	61.9±10.2	<0.001
Gender			<0.001
Male	125 (46)	201 (63)	
Female	147 (54)	119 (37)	
Other	2 (0.7)	0 (0)	
Ethnicity / race			<0.001
White	241 (88)	287 (90)	
Other	26 (9.5)	7 (2.2)	
Asian	3 (1.1)	16 (5.0)	
Black	4 (1.5)	10 (3.1)	
Employment			<0.001
Working/studying	205 (75)	121 (38)	
Not working / not studying	23 (8.4)	35 (11)	
Retired	46 (17)	164 (51)	
Highest level of education achieved			<0.001
College, undergraduate degree	120 (44)	111 (35)	
Postgraduate degree (Masters/PhD/MBA)	70 (26)	36 (11)	
Secondary school or high school	66 (24)	102 (32)	
Other	14 (5.1)	35 (11)	
Primary school	4 (1.5)	36 (11)	
Country			<0.001
UK	153 (56)	128 (40)	
the Netherlands	34 (12)	98 (31)	
Austria	33 (12)	53 (17)	
Denmark	30 (11)	37 (12)	
France	24 (8.8)	4 (1.3)	
Clinical characteristics			
Diabetes duration, years	23.8±15.6	20.4±8.9	0.14
Impaired awareness (Gold score ≥4)	58 (21)	86 (27)	0.11
Mean % time in range (≥3.9 mmol/l, ≤10 mmol/l)	61.0±15.1	64.8±20.7	<0.001
Mean % time above range (>10 mmol/l)	33.1±16.4	32.7±21.5	0.2
Mean % time below range (<3.9 mmol/l)	5.9±5.0	2.5±3.0	<0.001
Usual means of glucose monitoring			<0.001
Capillary blood glucose monitoring only (fingerprick)	67 (24)	188 (59)	
CGM without alerts	186 (68)	127 (40)	
CGM with alerts	21 (7.7)	5 (1.6)	
HbA_1c_, mmol/mol	56.5±9.5^b^	60.2±14.2^c^	0.009
HbA_1c_, %	7.3±0.9^b^	7.7±1.3^c^	0.009
Psychological characteristics			
Anxiety symptom			0.3
None (GAD-7 score <5)	176 (64)	195 (61)	
Mild (GAD-7 score 5–10)	69 (25)	77 (24)	
Moderate-to-severe (≥10)	29 (11)	48 (15)	
Depression symptoms			0.030
None (PHQ-9 score <5)	161 (59)	166 (52)	
Mild (PHQ-9 score 5–10)	73 (27)	80 (25)	
Moderate-to-severe (PHQ-9 score ≥10)	40 (15)	74 (23)	
Diabetes-specific QoL, DIDP composite score^d^	4.6±0.8	4.5±1.0	0.5
Cognitive functioning, PDQ-20 total score^e^	18.7±12.8	23.4±16.1	0.001
Fear of hypoglycaemia, HFS-II total score^f^	32.4±20.9	29.0±21.9	0.005
Severe diabetes distress (PAID-20 score ≥40)^g^	50 (18)	71 (22)	
Hypo-METRICS app completion			
Completion, morning check-in, percentage	86.1±12.7	86.5±12.3	0.6
Completion, evening check-in, percentage	89.8±11.4	91.6±9.8	0.005

Data are mean ± SD or *n* (%)

^a^Wilcoxon rank sum test; Fisher’s exact test; Pearson’s χ^2^ test

^b^Three values were missing

^c^One value was missing

^d^Higher score indicates greater negative impact across global life dimensions

^e^Higher score indicates greater perceived cognitive difficulties

^f^Higher score indicates higher fear of hypoglycaemia

^g^PAID-20 scores above 40 indicate severe diabetes distress

DIDP, Dawn Impact of Diabetes Profile; GAD-7, General Anxiety Disorder-7 questionnaire; HFS-II, Hypoglycaemia Fear Survey II; PAID, Problem Areas In Diabetes; PHQ-9, Patient Health Questionnaire-9

### Daily functioning check-ins

Across 594 participants and 70 days of app use, there was a potential maximum of 41,580 morning or evening check-ins. Participants with type 1 diabetes and type 2 diabetes respectively completed 86.1±12.7% and 86.5±12.3% (mean ± SD) of their morning check-ins and 89.8±11.4% and 91.6±9.8% (mean ± SD) of their evening check-ins.

### Distribution of hypoglycaemia (PRH and SDH)

A total of 32,519 night-time and 33,972 daytime intervals had PRH data, and valid (i.e. ≥70%) SDH data available (ESM Table 3). Of these, 72% and 64% were coded with no hypoglycaemia, 3.2% and 9.2% with PRH only, 17% and 11% with SDH only, and 7.8% and 16% with PRH and SDH, for the night-time and daytime intervals, respectively. As seen in ESM Table 3, the distribution of hypoglycaemia was significantly different between people with type 1 and type 2 diabetes.

### PRH, SDH and subjective daily functioning

**Fig. 1:**
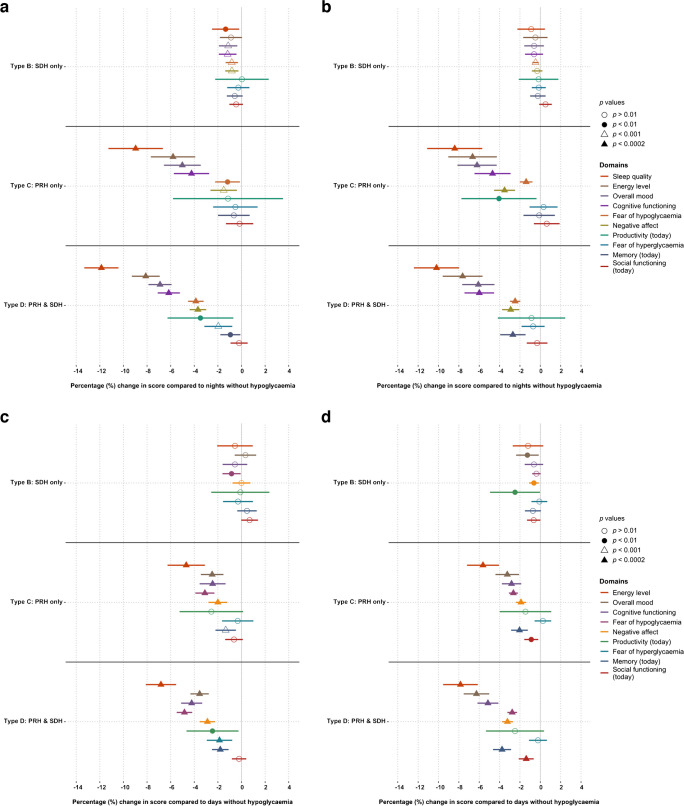
Effect of night-time hypoglycaemia (**a**, **b**) or daytime hypoglycaemia (**c**, **d**) among adults with type 1 diabetes (**a**, **c**,* n*=274) or type 2 diabetes (**b**, *n*=318; **d**, *n*=320). PRH episodes were reported in the app check-ins. SDH episodes (glucose levels <3.9 mmol/l for ≥15 min) were detected by (blinded) sensor. Results are coefficients from regression model adjusted for demographic, clinical, psychological and app-related factors. Higher scores on all scales represent ‘better’ daily functioning. Nights (**a**, **b**) or days (**c**, **d**) without hypoglycaemia (type A, at 0%) are used as reference. Domains are sorted by most to least impacted domain under type D (PRH and SDH) in (**a**). Lines represent 95% CIs (missing if going outside axis limit)

The associations between night-time PRH/SDH types (types A–D) and morning functioning are presented in Fig. 1a,b. For participants with type 1 and type 2 diabetes, regression coefficients for ‘sleep quality’, ‘energy level’, ‘overall mood’, ‘cognitive functioning’, ‘fear of hypoglycaemia later that day’ and ‘negative affect’, as well as ‘memory’ for participants with type 2 diabetes, were significantly lower (reflecting worse functioning) after nights with a PRH (type C and/or D) vs nights without hypoglycaemia (type A). The largest effect was seen for ‘sleep quality’, with >10% reduction in scores on nights with PRH and SDH (type D) compared with nights without (type A). For nights with only SDH (type B), there were small decreases but no statistically significant changes in scores on any domains compared with nights without hypoglycaemia (type A).

A similar pattern was shown for associations between daytime hypoglycaemia and evening functioning (Fig. 1c,d); however, PRH with SDH (type D) was additionally associated with significant lower scores (i.e. worsening) for ‘fear of hyperglycaemia while asleep’ and ‘memory’ in people with type 1 diabetes and ‘social functioning’ in people with type 2 diabetes. The domain with the largest reduction in scores for daytime hypoglycaemia was ‘energy level’. As for the night-time hypoglycaemia, no significant changes in scores for any of the domains was observed for participants with daytime SDH only (type B).

### PRH subtypes and subjective daily functioning

**Fig. 2:**
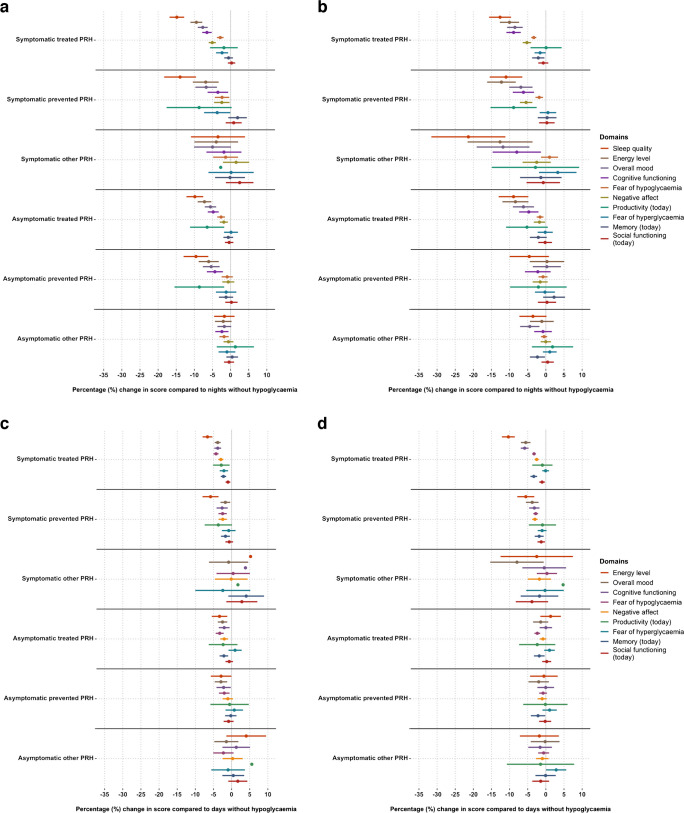
Effect of night-time (**a**, **b**) or daytime (**c**, **d**) PRH subtypes among adults with type 1 diabetes (**a**, **c**,* n*=274) or type 2 diabetes (**b**, *n*=318; **d**, *n*=320). PRH: episodes were reported in the app check-ins. Results are coefficients from regression model adjusted for SDH, baseline demographic, clinical, psychological and app-related factors. Higher scores on all scales represent ‘better’ daily functioning. Nights or days without hypoglycaemia (0%) are used as reference. Domains are sorted by most to least impacted domain under type D (PRH and SDH) in Fig. 1a. Lines represent 95% CIs (missing if going outside axis limit)

The exploratory analyses showing associations between PRH subtypes and morning and evening functioning are presented in Fig. 2.

For adults with type 1 diabetes and adults with type 2 diabetes, considering night-time ‘PRH detection’ (i.e. how hypoglycaemia is detected, see earlier), both symptomatic and asymptomatic PRH were associated with reduced scores on several domains of functioning at morning check-in, when compared with nights without PRH (Fig. 2a,b). Nights with symptomatic PRH were in most cases followed by a larger reduction in functioning compared with nights with asymptomatic PRH. Similarly, both symptomatic and asymptomatic daytime PRH (vs no PRH) were associated with reduced functioning at evening check-in (Fig. 2c,d) but generally with smaller effect size than seen for night-time hypoglycaemia. CIs for people with type 2 diabetes were often wider than those for people with type 1 diabetes, which may reflect fewer PRH episodes reported.

Similarly, Fig. 2a–d shows that, for ‘PRH management’ (i.e. how hypoglycaemia is managed, see earlier), treated episodes are generally (with some exceptions) associated with greater negative impact on daily functioning than prevented episodes, and prevented episodes more so than those categorised as ‘other’. Overall, the effect size appears larger for night-time PRH (Fig. 2a,b) than daytime PRH (Fig. 2c,d). Finally, although the CIs are wide, people with type 2 diabetes appear to be more impacted by night-time ‘symptomatic other’ PRH than people with type 1 diabetes.

### SDH subtypes and daily functioning

**Fig. 3:**
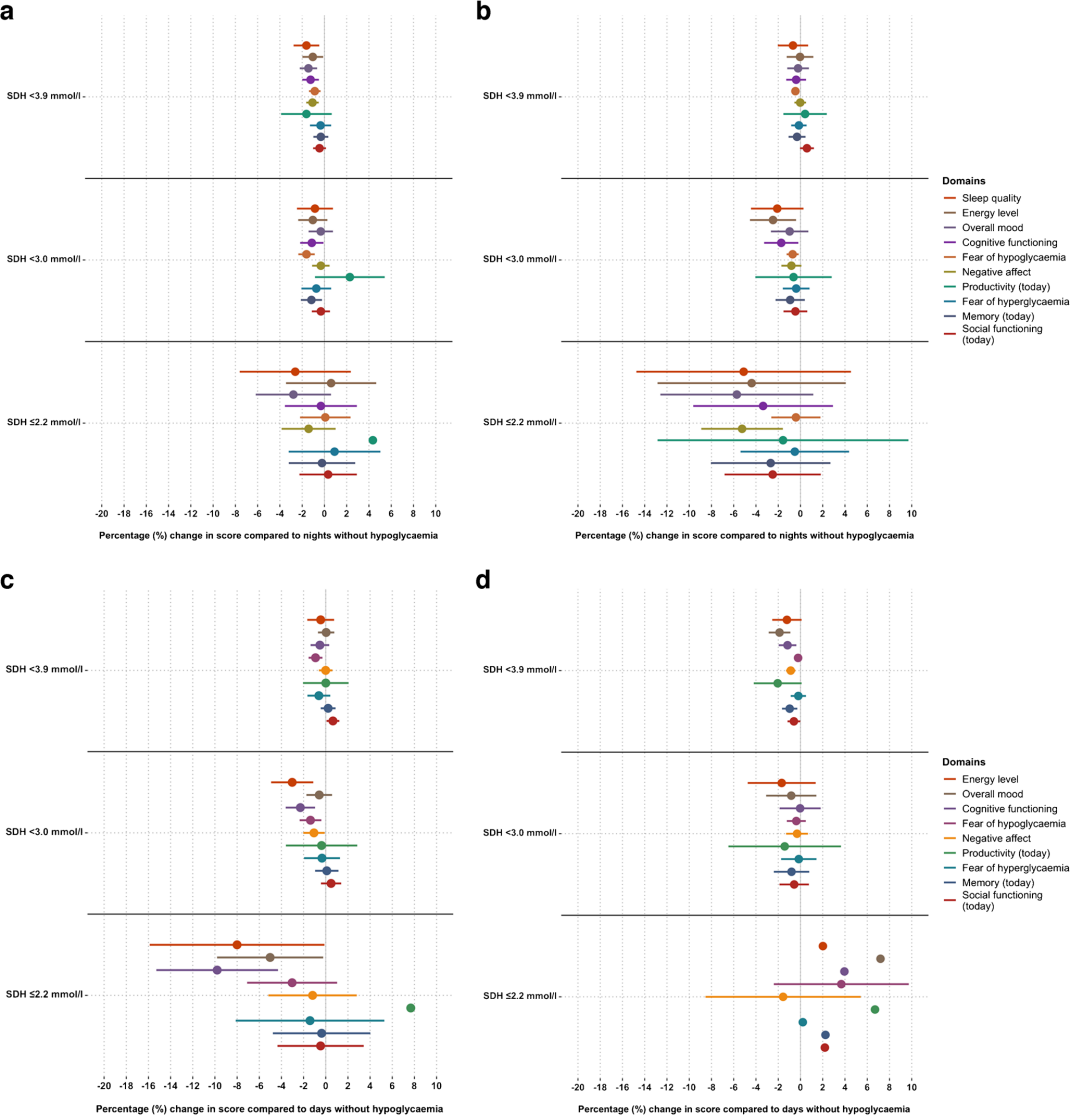
Effect of night-time (**a**, **b**) or daytime (**c**, **d**) SDH subtypes among adults with type 1 diabetes (**a**, **c**,* n*=274) or type 2 diabetes (**b**, *n*=318; **d**, *n*=320). SDH was detected by (blinded) sensor. Results are coefficients from regression model adjusted for PRH, baseline demographic, clinical, psychological and app-related factors. Higher scores on all scales represent ‘better’ daily functioning. Nights or days without hypoglycaemia (0%) are used as reference. Domains are sorted by most to least impacted domain under type D (PRH and SDH) in Fig. 1a. Lines represent 95% CIs (missing if going outside axis limit)

Figure 3 shows the exploratory analyses of the associations between SDH subtypes (i.e. lowest glucose levels in each time interval) and morning and evening functioning. When adjusted for PRH, SDH subtypes overall appeared to have minimal or no association with daily functioning domains. For SDH_3.9_ and SDH_3.0_, the majority of the effect sizes are close to 0% with small CIs. In contrast, for SDH_2.2_ there is more variation in estimates (wider CIs) with some effect sizes indicating possible worsening (e.g. for ‘overall mood’ from daytime episodes in people with type 1 diabetes, Fig. 3c), while others indicate possible improvement (e.g. for ‘fear of hypoglycaemia later that day’ from daytime episodes in people with type 2 diabetes, Fig. 3d).

## Discussion

In this large, 10 week observational study, adults with type 1 diabetes and insulin-treated type 2 diabetes undertook meticulous thrice-daily reporting of their daily functioning using the Hypo-METRICS app and wore blinded CGM. These novel data show that adults with type 1 or type 2 diabetes experienced a significant impact of PRH on their daily functioning, while SDH alone had no or minimal impact. Specifically, following night-time and daytime PRH, both participants with type 1 and type 2 diabetes reported worsening of ‘energy’, ‘mood’, ‘cognitive functioning’, ‘negative affect’ and ‘fear of hypoglycaemia later that day/while asleep’ (vs days/nights without PRH and SDH). In addition, night-time PRH was significantly associated with worsening of ‘sleep quality’ (for type 1 and type 2 diabetes) and ‘memory’ (for type 2 diabetes). Daytime PRH was also associated with worsening of ‘fear of hyperglycaemia while asleep’ (for type 1 diabetes), ‘memory’ (for type 1 and type 2 diabetes) and ‘social functioning’ (for type 2 diabetes). The changes in domain scores were only significant if a PRH was reported (type C and/or D), while changes following SDH alone (type B) did not reach statistical significance for either type of diabetes. Exploratory analyses showed that, overall, subtypes of PRH (symptomatic vs asymptomatic, and treated vs prevented vs other) were associated with reduced daily functioning when compared with days or nights without PRH. Night-time PRH, presence of symptoms and hypoglycaemia categorised as treated (rather than prevented) generally lead to larger reductions in functioning. In contrast, subtypes of SDH (SDH_3.9_, SDH_3.0_ and SDH_2.2_) overall had minimal associations with daily functioning.

To our knowledge, no published studies provide as extensive a collection of SDH and PRH as the current study (70 days), enabling prospective assessments of their unique associations with daily functioning. In a recent qualitative study, people with type 1 diabetes reported how hypoglycaemia impacted several aspects of their QoL, such as mental health, sleep, leisure activities, work and social life [23]. Another study focusing on the impact of self-reported hypoglycaemia over the past 30 days in people with type 2 diabetes found that self-treated hypoglycaemia was negatively associated with participants’ well-being and functioning, including cognitive functioning, leisure activities, social life and work [24]. The multidimensional day-to-day impacts shown in the current study align with these broader impacts.

Recent prospective studies have assessed the impact of daily hypoglycaemia with mixed results. Preliminary results from an ‘ecological momentary assessment’ (EMA) study including people with type 1 diabetes found that SDH affected energy but not mood [25]. Another study found no effect of night-time SDH on self-reported mood or effectiveness at work the next day but found an improvement in self-reported ‘health status’ in people with type 1 diabetes and impaired awareness [12, 26]. A recent study by Polonsky and Fortmann also reported no significant associations between hypoglycaemia and daily mood [27]. Wagner et al found that SDH was associated with lower positive affect in people with type 2 diabetes [28]. Although some of these studies report on whether participants had symptoms, it remains unclear whether episodes without symptoms were recognised via participants’ usual glucose monitors. The current study indicates that it may only be the episodes that a person recognises (via symptoms or glucose monitoring) that impact on daily functioning. Analyses of SDH subtypes, adjusted for PRH, showed that the associations between PRH and daily functioning are unlikely to only be due to lower glucose during PRHs. Further, the current study provided a more intensive data-collection across more domains of daily functioning compared with previous studies, possibly explaining why results only partially align with results of prior EMA studies.

High ecological (i.e. ‘real-world’) validity is a central strength of the current study. Numerous hypoglycaemic clamp studies have demonstrated that acute hypoglycaemia impairs multiple domains of cognitive functioning, mood and emotions [29–31]. The ecological validity of these experimentally induced impairments is unclear [32, 33]. However, in real-world settings, many factors can influence the relationship between hypoglycaemia and daily functioning, including the burden of checking and tracking glucose values, the food and activity planning required to prevent hypoglycaemia (with implications for spontaneity) and the fear of episodes leading to socially embarrassing or dangerous situations, all of which have been highlighted in qualitative research [23]. Importantly, this study has provided real-world evidence of the impact of hypoglycaemia on self-reported cognitive functioning.

There is a risk that the most impactful episodes are not recorded in the app as priority to treat, or the direct impact (e.g. concentration difficulties) from episodes may have influenced the ability to complete check-ins. The relatively wide time intervals (6 h) to enter each check-in may have led participants to delay completion to a more convenient time, potentially biasing responses. The high average completion rates suggest that participants were highly motivated and more work is needed to explore predictors of completion to understand whether certain characteristics of the participants are over- or under-represented in this study. Knowing that this study focused on the impact of hypoglycaemia may have prompted participants to pay more attention to how hypoglycaemia influenced them, compared with usual everyday life, or may have led to an over-representation of participants with hypoglycaemia-related problems. Reassuringly, a preliminary analysis of data from the Hypo-METRICS cohort suggests no significant impact on hypoglycaemia reporting during the course of the study. The lower frequency of SDH_2.2_ makes it challenging to draw firm conclusions about the effects of these low glucose levels and more work is needed. Importantly, this study was limited to five European countries and there was little ethnic diversity. Further studies are needed in non-European and ethnically diverse populations.

Our data has several clinical implications. While CGM offers considerable advantages in terms of minimising hypoglycaemia, optimising overall glucose levels and improving diabetes-specific QoL [34, 35], the current study highlights some potential disadvantages. The finding that SDH from the blinded CGM (i.e. episodes not recognised by the person) had minimal impact on daily functioning but asymptomatic PRH (i.e. episodes recognised via glucose measurement) did, suggests that people with diabetes might be resilient to the direct effect of biochemical (unrecognised) hypoglycaemia. However, it also suggests that healthcare professionals need to be aware of the negative consequences of being alerted to asymptomatic low glucose. Although recurrent exposure to hypoglycaemia may increase the risk of impaired awareness and severe hypoglycaemia [36], raising the glucose threshold for a sensor alert might increase the frequency of alarms and thus negatively affect daily functioning. Further, the added burden of symptomatic (compared with asymptomatic) episodes, and the fact that both treating and preventing episodes has negative impact on daily functioning, warrants attention in clinical practice and future interventions. The current study suggests that the distinction between SDH and PRH, and whether the episodes are recognised by the individual, is important when assessing the impact of hypoglycaemia. It also suggests that use of CGM alone to determine the impact of an intervention to reduce hypoglycaemia may not be adequate. PRH needs to be reported as well, as this is what is meaningful to a person in terms of its impact on their daily functioning. We cannot make conclusions about the exact mechanisms that link recognised hypoglycaemia with impaired daily functioning. The authors hypothesise that the disruptive impact of recognising and counteracting falling glucose on sleep and usual routines (e.g. work and socialising) may be important factors.

There are several avenues for future research into the day-to-day impact of hypoglycaemia using EMA, which is becoming a common method in diabetes research [37–39]. First, despite statistically significant associations, further work is needed to understand whether observed changed are important and meaningful for the person living with diabetes [40]. Second, although the present analysis controlled for several person-related characteristics, the results represent means across participants. Thus, future analyses, such as cluster analyses or mixture modelling, could explore whether there are subgroups of people particularly vulnerable to the impact of hypoglycaemia [41]. Third, the current study focused on presence or absence of hypoglycaemia but it would be useful to examine the cumulative impact of multiple episodes of hypoglycaemia as well as the relationship between the duration and/or depth of a single episode of hypoglycaemia and the extent and duration of its impact on daily functioning. Understanding the cumulative impact also becomes relevant when considering that more episodes of PRH were reported by participants with type 1 diabetes, compared with those with type 2 diabetes (perhaps due to the higher proportion of CGM users). Finally, while the current analyses were adjusted for level of awareness, more work is needed to investigate whether hypoglycaemia awareness status is an effect modifier.

In conclusion, this study provides novel insights into the subjective daily functioning of adults with type 1 diabetes and insulin-treated type 2 diabetes following daytime and night-time hypoglycaemia episodes. The observed reductions in daily functioning following hypoglycaemia are explained, principally, by episodes recognised by the participant (i.e. PRH with or without SDH), either from symptoms or via their glucose monitoring device and may be related to actions required to avoid or treat low glucose levels. The (blinded) SDH had limited contribution. These findings emphasise the need for researchers and clinicians to consider the burden on people with insulin-treated diabetes not only in experiencing low glucose levels but also in recognising, managing and avoiding episodes of hypoglycaemia, and indicate a need for critical review of the importance to the wearer of hypoglycaemia episodes that are only detected by the sensor.

## Supplementary Information

Below is the link to the electronic supplementary material.

Supplementary file1 (PDF 502 KB)

## Data Availability

The data underlying the results presented in the study are available from the Hypo-RESOLVE data repository for researchers who meet the criteria for access. Please contact Hypo-RESOLVE for further details (chair of the publication committee Professor Stephanie Amiel or principal investigator Professor Pratik Choudhary; https://hypo-resolve.eu/contact).

## Abbreviations

CGM: Continuous glucose monitoring
EMA: Ecological momentary assessment
Hypo-METRICS: Hypoglycaemia MEasurement, ThResholds and ImpaCtS
Hypo-RESOLVE: Hypoglycaemia – Redefining SOLutions for better liVEs
PRH: Person-reported hypoglycaemia
QoL: Quality of life
SDH: Sensor-detected hypoglycaemia

## References

[1] Frier BM (2014) Hypoglycaemia in diabetes mellitus: epidemiology and clinical implications. Nat Rev Endocrinol 10(12):711–722. 10.1038/nrendo.2014.170 25287289 10.1038/nrendo.2014.170

[2] Fidler C, Elmelund Christensen T, Gillard S (2011) Hypoglycemia: an overview of fear of hypoglycemia, quality-of-life, and impact on costs. J Med Econ 14(5):646–655. 10.3111/13696998.2011.610852 21854191 10.3111/13696998.2011.610852

[3] Matlock KA, Broadley M, Hendrieckx C et al (2022) Changes in quality of life following hypoglycaemia in adults with type 2 diabetes: a systematic review of longitudinal studies. Diabet Med 39(1):e14706. 10.1111/dme.14706 34596292 10.1111/dme.14706 PMC9293422

[4] Chatwin H, Broadley M, Speight J et al (2021) The impact of hypoglycaemia on quality of life outcomes among adults with type 1 diabetes: a systematic review. Diabetes Res Clin Pract 174:108752. 10.1016/j.diabres.2021.108752 33722700 10.1016/j.diabres.2021.108752

[5] Khunti K, Alsifri S, Aronson R et al (2016) Rates and predictors of hypoglycaemia in 27 585 people from 24 countries with insulin-treated type 1 and type 2 diabetes: the global HAT study. Diabetes Obes Metab 18(9):907–915. 10.1111/dom.12689 27161418 10.1111/dom.12689 PMC5031206

[6] Danne T, Nimri R, Battelino T et al (2017) International consensus on use of continuous glucose monitoring. Diabetes Care 40(12):1631. 10.2337/dc17-1600 29162583 10.2337/dc17-1600 PMC6467165

[7] Divilly P, Martine-Edith G, Mahmoudi Z et al (2023) 250-OR: majority of sensor hypoglycemia is not detected by people living with diabetes—hypo-METRICS study. Diabetes 72(Supplement_1). 10.2337/db23-250-OR

[8] Chatwin H, Broadley M, Hendrieckx C et al (2022) The impact of hypoglycaemia on quality of life among adults with type 1 diabetes: results from “YourSAY: Hypoglycaemia”. J Diabetes Complications 37(11):108232. 10.1016/j.jdiacomp.2022.108232 10.1016/j.jdiacomp.2022.108232 35927177

[9] Amiel SA (2021) The consequences of hypoglycaemia. Diabetologia 64(5):963–970. 10.1007/s00125-020-05366-3 33550443 10.1007/s00125-020-05366-3 PMC8012317

[10] Hendrieckx C, Ivory N, Singh H, Frier BM, Speight J (2019) Impact of severe hypoglycaemia on psychological outcomes in adults with Type 2 diabetes: a systematic review. Diabet Med 36(9):1082–1091. 10.1111/dme.14067 31271669 10.1111/dme.14067

[11] Blome C, Augustin M (2015) Measuring change in quality of life: bias in prospective and retrospective evaluation. Value Health 18(1):110–115. 10.1016/j.jval.2014.10.007 25595241 10.1016/j.jval.2014.10.007

[12] Henriksen MM, Andersen HU, Thorsteinsson B, Pedersen-Bjergaard U (2021) Effects of continuous glucose monitor-recorded nocturnal hypoglycaemia on quality of life and mood during daily life in type 1 diabetes. Diabetologia 64(4):903–913. 10.1007/s00125-020-05360-9 33443591 10.1007/s00125-020-05360-9

[13] Søholm U, Broadley M, Zaremba N et al (2022) Investigating the day-to-day impact of hypoglycaemia in adults with type 1 or type 2 diabetes: design and validation protocol of the Hypo-METRICS application. BMJ Open 12(2):e051651. 10.1136/bmjopen-2021-051651 35105572 10.1136/bmjopen-2021-051651 PMC8808414

[14] Søholm U, Broadley M, Zaremba N et al (2023) Psychometric properties of an innovative smartphone application to investigate the daily impact of hypoglycemia in people with type 1 or type 2 diabetes: The Hypo-METRICS app. PloS One 18(3):e0283148. 10.1371/journal.pone.0283148 36930585 10.1371/journal.pone.0283148 PMC10022775

[15] de Galan BE, McCrimmon RJ, Ibberson M et al (2020) Reducing the burden of hypoglycaemia in people with diabetes through increased understanding: design of the Hypoglycaemia REdefining SOLutions for better liVEs (Hypo-RESOLVE) project. Diabet Med 37(6):1066–1073. 10.1111/dme.14240 31970814 10.1111/dme.14240 PMC7317819

[16] Divilly P, Zaremba N, Mahmoudi Z et al (2022) Hypo-METRICS: Hypoglycaemia - MEasurement, ThResholds and ImpaCtS - A multi-country clinical study to define the optimal threshold and duration of sensor-detected hypoglycaemia that impact the experience of hypoglycaemia, quality of life and health economic outcomes: the study protocol. Diabet Med 39(9):e14892. 10.1111/dme.14892 35633291 10.1111/dme.14892 PMC9542005

[17] Gold AE, MacLeod KM, Frier BM (1994) Frequency of severe hypoglycemia in patients with type I diabetes with impaired awareness of hypoglycemia. Diabetes Care 17(7):697–703. 10.2337/diacare.17.7.697 7924780 10.2337/diacare.17.7.697

[18] Søholm U, Zaremba N, Broadley M et al (2023) Assessing the content validity, acceptability, and feasibility of the hypo-METRICS app: survey and interview study. JMIR Diabetes 8:e42100. 10.2196/42100 37773626 10.2196/42100 PMC10576226

[19] Battelino T, Danne T, Bergenstal RM et al (2019) Clinical targets for continuous glucose monitoring data interpretation: recommendations from the international consensus on time in range. Diabetes Care 42(8):1593–1603. 10.2337/dci19-0028 31177185 10.2337/dci19-0028 PMC6973648

[20] Raccah D, Sulmont V, Reznik Y et al (2009) Incremental value of continuous glucose monitoring when starting pump therapy in patients with poorly controlled type 1 diabetes: the RealTrend study. Diabetes Care 32(12):2245–2250. 10.2337/dc09-0750 19767384 10.2337/dc09-0750 PMC2782985

[21] (2022) RStudio Team (2022) RStudio: Integrated Development for R. RStudio Version: 2022.2.1.461, PBC, Boston, MA. Available from http://www.rstudio.com/

[22] Koller M (2016) robustlmm: an R package for robust estimation of linear mixed-effects models. J Stat Softw 75(6):1–24 32655332 10.18637/jss.v075.i01 PMC7351245

[23] Chatwin H, Broadley M, Valdersdorf Jensen M et al (2021) “Never again will I be carefree”: a qualitative study of the impact of hypoglycemia on quality of life among adults with type 1 diabetes. BMJ Open Diabetes Res Care 9(1):e002322. 10.1136/bmjdrc-2021-002322 34400465 10.1136/bmjdrc-2021-002322 PMC8370551

[24] Brod M, Rana A, Barnett AH (2012) Impact of self-treated hypoglycaemia in type 2 diabetes: a multinational survey in patients and physicians. Curr Med Res Opin 28(12):1947–1958. 10.1185/03007995.2012.743457 23150950 10.1185/03007995.2012.743457

[25] Ehrmann D, Schmitt AJ, Rubertus P, Kulzer B, Hermanns N (2020) 783-P: can mood and energy levels be predicted by preceding glucose values? Combining Ecological Momentary Assessment (EMA) and Continuous Glucose Monitoring (CGM). Diabetes 69(Supplement 1):783-P. 10.2337/db20-783-P

[26] Søholm U, Broadley MM, Choudhary P et al (2021) Does nocturnal hypoglycaemia really improve quality of life? Diabetologia 64(8):1893–1894. 10.1007/s00125-021-05475-7 34014372 10.1007/s00125-021-05475-7

[27] Polonsky WH, Fortmann AL (2020) The influence of time in range on daily mood in adults with type 1 diabetes. J Diabetes Complications 34(12):107746. 10.1016/j.jdiacomp.2020.107746 33077350 10.1016/j.jdiacomp.2020.107746

[28] Wagner J, Armeli S, Tennen H, Bermudez-Millan A, Wolpert H, Pérez-Escamilla R (2017) Mean levels and variability in affect, diabetes self-care behaviors, and continuously monitored glucose: a daily study of Latinos with type 2 diabetes. Psychosom Med 79(7):798–805. 10.1097/psy.0000000000000477 28437381 10.1097/PSY.0000000000000477 PMC5573602

[29] Inkster B, Frier B (2012) The effects of acute hypoglycaemia on cognitive function in type 1 diabetes. Br J Diabetes Vasc Dis 12:221–226. 10.1177/1474651412466273

[30] Merbis MA, Snoek FJ, Kanc K, Heine RJ (1996) Hypoglycaemia induces emotional disruption. Patient Educ Couns 29(1):117–122. 10.1016/0738-3991(96)00940-8 9006228 10.1016/0738-3991(96)00940-8

[31] Warren RE, Zammitt NN, Deary IJ, Frier BM (2007) The effects of acute hypoglycaemia on memory acquisition and recall and prospective memory in type 1 diabetes. Diabetologia 50(1):178–185. 10.1007/s00125-006-0535-6 17143604 10.1007/s00125-006-0535-6

[32] Toplak ME, West RF, Stanovich KE (2013) Practitioner review: do performance-based measures and ratings of executive function assess the same construct? J Child Psychol Psychiatry 54(2):131–143. 10.1111/jcpp.12001 23057693 10.1111/jcpp.12001

[33] Heise T, Zijlstra E, Nosek L, Heckermann S, Plum-Mörschel L, Forst T (2016) Euglycaemic glucose clamp: what it can and cannot do, and how to do it. Diabetes Obes Metab 18(10):962–972. 10.1111/dom.12703 27324560 10.1111/dom.12703

[34] Polonsky WH, Hessler D, Ruedy KJ, Beck RW (2017) The impact of continuous glucose monitoring on markers of quality of life in adults with type 1 diabetes: further findings from the DIAMOND randomized clinical trial. Diabetes Care 40(6):736–741. 10.2337/dc17-0133 28389582 10.2337/dc17-0133

[35] Riddlesworth T, Price D, Cohen N, Beck RW (2017) Hypoglycemic event frequency and the effect of continuous glucose monitoring in adults with type 1 diabetes using multiple daily insulin injections. Diabetes Ther Res Treat Educ Diabetes Relat Disord 8(4):947–951. 10.1007/s13300-017-0281-4 10.1007/s13300-017-0281-4 PMC5544617 28616804

[36] Tesfaye N, Seaquist ER (2010) Neuroendocrine responses to hypoglycemia. Ann N Y Acad Sci 1212:12–28. 10.1111/j.1749-6632.2010.05820.x 21039590 10.1111/j.1749-6632.2010.05820.x PMC2991551

[37] Zink J, Nicolo M, Imm K et al (2020) Interstitial glucose and subsequent affective and physical feeling states: a pilot study combining continuous glucose monitoring and ecological momentary assessment in adolescents. J Psychosom Res 135:110141. 10.1016/j.jpsychores.2020.110141 32447156 10.1016/j.jpsychores.2020.110141 PMC7452157

[38] Inada S, Iizuka Y, Ohashi K et al (2019) Preceding psychological factors and calorie intake in patients with type 2 diabetes: investigation by ecological momentary assessment. BioPsychoSocial medicine 13:20. 10.1186/s13030-019-0161-4 31508145 10.1186/s13030-019-0161-4 PMC6727338

[39] Merwin RM, Dmitrieva NO, Honeycutt LK et al (2015) Momentary predictors of insulin restriction among adults with type 1 diabetes and eating disorder symptomatology. Diabetes Care 38(11):2025–2032. 10.2337/dc15-0753 26384389 10.2337/dc15-0753 PMC4876774

[40] de Vet HC, Terwee CB, Ostelo RW, Beckerman H, Knol DL, Bouter LM (2006) Minimal changes in health status questionnaires: distinction between minimally detectable change and minimally important change. Health Qual Outcomes 4:54–54. 10.1186/1477-7525-4-54 10.1186/1477-7525-4-54 PMC1560110 16925807

[41] Pina A, Macedo MP, Henriques R (2020) Clustering clinical data in R. Methods Mol Biol 2051:309–343. 10.1007/978-1-4939-9744-2_14 31552636 10.1007/978-1-4939-9744-2_14

