# Supplementary material for: The impact of hypoglycaemia on daily functioning among adults with diabetes: a prospective observational study using the Hypo-METRICS app
Source: Diabetologia. 2024 Jul 30;67(10):2160–74. doi: 10.1007/s00125-024-06233-1 (PMC11447150; doi:10.1007/s00125-024-06233-1)
Supplement: Supplementary file 1 — Supplementary file1 (PDF 502 KB) [file 125_2024_6233_MOESM1_ESM.pdf]

Electronic supplementary material:

Overview:

- **Page 2:** ESM Table 1: Overview of Hypo-METRICS app daily functioning content
- **Page 5:** ESM Table 2: Control variables used in adjusted models
- **Page 6:** ESM Table 3: Distribution of PRH and SDH
- **Page 8:** ESM Figure 1: Categorisation of person-reported hypoglycaemia (PRH)

ESM Table 1: Overview of Hypo-METRICS app daily functioning content

**The three daily check-ins**					
**Module names[1] and items**	**Response option**	**Domains[2]**	**Completion timepoints ('Check-ins')**		
			**Morning**	**Afternoon**	**Evening**
Sleep quality module (2 items)					
1. *How well did you sleep?*	*Extremely badly (0) – Extremely well (10)*	*Sleep quality (1/2)*	*x*		
2. *When you woke up how did you feel?*	*Extremely tired (0) – Extremely rested (10)*	*Sleep quality (2/2)*	*x*		
General well-being module (7 items)					
3. *How is your mood right now?*	*Extremely bad (0) – Extremely good (10)*	*Overall mood (1/1)*	*x*	*x*	*x*
4. *How anxious do you feel right now?[3]*	*Not at all (0) - Extremely (10)*	*Negative affect (1/2)*	*x*	*x*	*x*
5. *How irritable do you feel right now?[3]*	*Not at all (0) - Extremely (10)*	*Negative affect (2/2)*	*x*	*x*	*x*
6. *How is your energy level right now?*	*Extremely low (0) – Extremely high (10)*	*Energy level (1/1)*	*x*	*x*	*x*
7. *How alert do you feel right now?*	*Not at all (0) - Extremely (10)*	*Cognitive functioning (1/2)*	*x*	*x*	*x*
8. *How well are you able to concentrate right now?*	*Not at all (0) - Extremely (10)*	*Cognitive functioning (2/2)*	*x*	*x*	*x*
8. *How easy was it for you to remember things today?*	*Not at all (0) - Extremely (10)*	*Memory (today) (1/1)*			*x*
Fear of hypo-/hyperglycaemia module (4 items)					
10. How worried are you about having a hypo later today?[3]	*Not at all (0) - Extremely (10)*	*Fear of hypoglycaemia later today (1/1)*	*x*	*x*	
11. How worried are you about having high blood glucose later today?[3]	*Not at all (0) - Extremely (10)*	*Fear of hyperglycaemia later today (1/1)*	*x*	*x*	
12. How worried are you about having a hypo while asleep?[3]	*Not at all (0) - Extremely (10)*	*Fear of hypoglycaemia while asleep (1/1)*			*x*
13. How worried are you about having high blood glucose while asleep?[3]	*Not at all (0) – Extremely (10)*	*Fear of hyperglycaemia while asleep (1/1)*			*x*
Social interactions module (1 item)					
14. How well did you get along with other people today?	*Extremely badly (0) – Extremely well (10)*	*Social functioning (1/1)*			*x*
Work and productivity module (4 items)					
15. How many hours did you work today?	*(Select hours / minute)*	*Hours worked[4] (1/1)*			*x*
16. How many hours did you miss from work for ANY reason today? [this includes health issues, vacation, holiday, etc.]	*(Select hours / minute)*	*Hours missed from work[4] (1/1)*			*x*
17. How many hours did you miss from activities other than work today for ANY reason (e.g. study, housework, shopping, family or leisure activities)?	*(Select hours / minute)*	*Hours missed from other activities than work[4] (1/1)*			*x*

18. How productive were you while working today?	*Extremely unproductive (0) – Extremely productive (10)*	*Productivity*[4]			*x*
Self-report of hypos while asleep module (8 items)					
19. During the night, did you have a hypo OR take action to prevent a hypo that was about to happen?	*Yes, No, Not sure*	*NA*	*x*		
20. How many hypos did you have?	*(Select number)*	*NA*	*x*		
21. At what time did this happen?	*(Select hour / minute)*	*NA*	*x*		
22. How did you detect your hypo or a hypo that was about to happen? (Select all that apply)	*- I had symptoms<br>- Someone told me they thought I might be having a hypo<br>- I did a finger prick check (SMBG)<br>- I checked my sensor<br>- My sensor alarmed<br>- I "just knew"<br>- I slept through it – and realised when I woke up<br>- Other:*	*NA*	*x*		
23. What happened? (Select all that apply)	*- I ate / drank to prevent a hypo<br>- I ate / drank to treat a hypo<br>- I reduced insulin<br>-Someone else gave me something to eat/drink<br>-Someone else gave me glucagon<br>-An ambulance was called<br>-I was admitted to hospital<br>-I took no action<br>-None of the above<br>-Skip questions*	*NA*	*x*		
24. Overall: How bothersome was hypoglycaemia for you last night?[5]	*Not at all (0) – Extremely (10)*	*NA*	*x*		
25. Overall: How much sleep did you lose due to hypoglycaemia?[5]	*(Select hour / minute)*	*NA*	*x*		
26. Overall: How worried were you about going back to sleep?[5]	*Not at all – Extremely (10)*	*NA*	*x*		
Self-report of daytime hypos module (7 items)					
27. Today, did you have a hypo OR take action to prevent a hypo that was about to happen?	*Yes, No, Not sure*	*NA*			*x*
20.1 How many hypos did you have?	*(Select number)*	*NA*			*x*
21.1 At what time did this happen?	*(Select hour / minute)*	*NA*			*x*
22.1 How did you detect your hypo or a hypo that was about to happen?	*- I had symptoms*	*NA*			*x*

	- Someone told me they thought I might be having a hypo<br>*- I did a finger prick check (SMBG)*<br>*- I checked my sensor*<br>*- My sensor alarmed*<br>*- I "just knew"*<br>*- Other:*				
23.1 What happened?	*- I ate / drank to prevent a hypo*<br>*- I ate / drank to treat a hypo*<br>*- I reduced insulin*<br>*-Someone else gave me something to eat/drink*<br>*-Someone else gave me glucagon*<br>*-An ambulance was called*<br>*-I was admitted to hospital*<br>*-I took no action*<br>*-None of the above*<br>*-Skip questions*	*NA*			*x*
28. Overall: How bothersome was hypoglycaemia for you today?[5]	*Not at all (0) – Extremely (10)*	*NA*			*x*
29. Overall: How long was it before you were feeling your "usual self" again?[5]	*(Select hour / minute)*	*NA*			*x*

[1]Module names refer to the titles used in the app when presenting the items (i.e., what the study participant would see)

[2]Domains refer to individual items or subscales (developed based on psychometric properties [1]) that was used for analysis; for example the domain 'Sleep quality' is a subscale consisting of two individual items, while the domain 'Overall mood' consist of just one item. Subscales were created taking a mean of the individual item scores.

[3]Scores on certain items (anxiety, irritability, and fear related items) were reversed, so that a higher score indicate improvement (i.e., less anxiety, irritability, or fear). This was to align that an increase in scores on all items would indicate improvement of daily functioning.

[4]Only the Productivity domain from the Work and productivity module were included for analysis in this paper (as the remaining items had a different response scale with hour and minute input; these will be included in other Hypo-RESOLVE publications focusing on health economic impact from hypoglycaemia).
[5]These items were not included in the current analysis (with comparison of days/nights with vs. without hypoglycaemia), as these items where only asked on days/nights with hypoglycaemia and therefore do not allow for a comparison with days/nights without hypoglycaemia.

ESM Table 2: Control variables used in adjusted models

**Baseline demographics**	Gender
	Age
**Clinical factors**	Duration of insulin therapy
	Impaired awareness of hypoglycaemia (Gold score)
	Time above range (in each time interval)
	Severe hypoglycaemia in past 12 months (baseline)
	Usual means of glucose monitoring (capillary blood glucose monitoring only (fingerprick), continuous glucose monitoring without alerts or continuous glucose monitoring with alerts)
**Psychological factors**	Anxiety symptoms baseline (GAD 7)
	Depressive symptoms baseline (PHQ-9)
	Diabetes-specific QoL baseline (DIDP)
	Cognitive functioning baseline (PDQ-20)
	Diabetes distress baseline (PAID 20)
	Hypoglycaemia fear baseline (HFS-II)
**Hypo-METRICS app completion**	Percentage number of morning and evening check-ins completed.

ESM Table 3: Distribution of PRH and SDH

	**Type 1**, N = 19,180[1]	**Type 2**, N = 22,400[1]	**p-value**[2]
Night-time hypoglycaemia			
Distribution of PRH and SDH, night-time			<0.001
Type A: PRH (-) | SDH (-)	9,435 (63%)	14,015 (79%)	
Type B: PRH (-) | SDH (+)	3,130 (21%)	2,383 (14%)	
Type C: PRH (+) | SDH (-)	542 (3.6%)	493 (2.8%)	
Type D: PRH (+) | SDH (+)	1,773 (12%)	748 (4.2%)	
(Missing)	4,300	4,761	
PRH subtypes, night-time			<0.001
No PRH	12,565 (85%)	16,398 (93%)	
Asymptomatic other PRH	379 (2.6%)	260 (1.5%)	
Asymptomatic prevented PRH	259 (1.7%)	142 (0.8%)	
Asymptomatic treated PRH	596 (4.0%)	216 (1.2%)	
Symptomatic other PRH	50 (0.3%)	32 (0.2%)	
Symptomatic prevented PRH	143 (1.0%)	160 (0.9%)	
Symptomatic treated PRH	815 (5.5%)	385 (2.2%)	
(Missing)	4,373	4,807	
SDH subtypes, night-time			<0.001
No SDH	11,351 (67%)	16,495 (83%)	
SDH <3.9 mmol/L	3,588 (21%)	2,645 (13%)	
SDH <3.0 mmol/L	1,771 (11%)	774 (3.9%)	
SDH ≤2.2 mmol/L	116 (0.7%)	43 (0.2%)	
(Missing)	2,354	2,443	
Daytime hypoglycaemia			
Distribution of PRH and SDH, daytime			<0.001
Type A: PRH (-) | SDH (-)	6,966 (45%)	14,758 (80%)	
Type B: PRH (-) | SDH (+)	2,339 (15%)	1,465 (7.9%)	
Type C: PRH (+) | SDH (-)	1,887 (12%)	1,248 (6.7%)	
Type D: PRH (+) | SDH (+)	4,233 (27%)	1,076 (5.8%)	
(Missing)	3,755	3,853	
PRH subtypes, daytime			<0.001

No PRH	9,305 (61%)	16,223 (88%)	
Asymptomatic other PRH	109 (0.7%)	92 (0.5%)	
Asymptomatic prevented PRH	461 (3.0%)	189 (1.0%)	
Asymptomatic treated PRH	948 (6.2%)	362 (2.0%)	
Symptomatic other PRH	39 (0.3%)	25 (0.1%)	
Symptomatic prevented PRH	801 (5.2%)	517 (2.8%)	
Symptomatic treated PRH	3,646 (24%)	1,094 (5.9%)	
(Missing)	3,871	3,898	
SDH subtypes, daytime			<0.001
No SDH	9,742 (58%)	17,294 (86%)	
SDH <3.9 mmol/L	5,358 (32%)	2,370 (12%)	
SDH <3.0 mmol/L	1,707 (10%)	348 (1.7%)	
SDH ≤2.2 mmol/L	59 (0.3%)	7 (<0.1%)	
(Missing)	2,314	2,381	

[1] n (%)

[2] Pearson's Chi-squared test

Please note: The submission times of each morning and evening check-in were used to divide each 24-hour period into night-time and daytime intervals. For each participant, each time-interval was categorised by presence (+) or absence (-) of PRH and/or SDH. Each time interval had valid data if information about PRH was reported in the app (i.e., non-missing), and ≥70% SDH data were available for that time-interval. The percentage in brackets above refers to the distribution of time-intervals with valid data. For example, when considering the “Distribution of PRH and SDH, night-time” for people with type 1 diabetes above: out of a total of 19,180 possible night-time intervals, 4,300 intervals had missing (non-valid) data, and of the remaining valid intervals, 63% of these were coded as Type A (no PRH or SDH recorded), 21% were coded as Type B (only SDH recorded), 3.6% were coded as Type C (only PRH recorded), and 12% coded as Type D (PRH and SDH).

ESM Figure 1: Categorisation of person-reported hypoglycaemia (PRH)

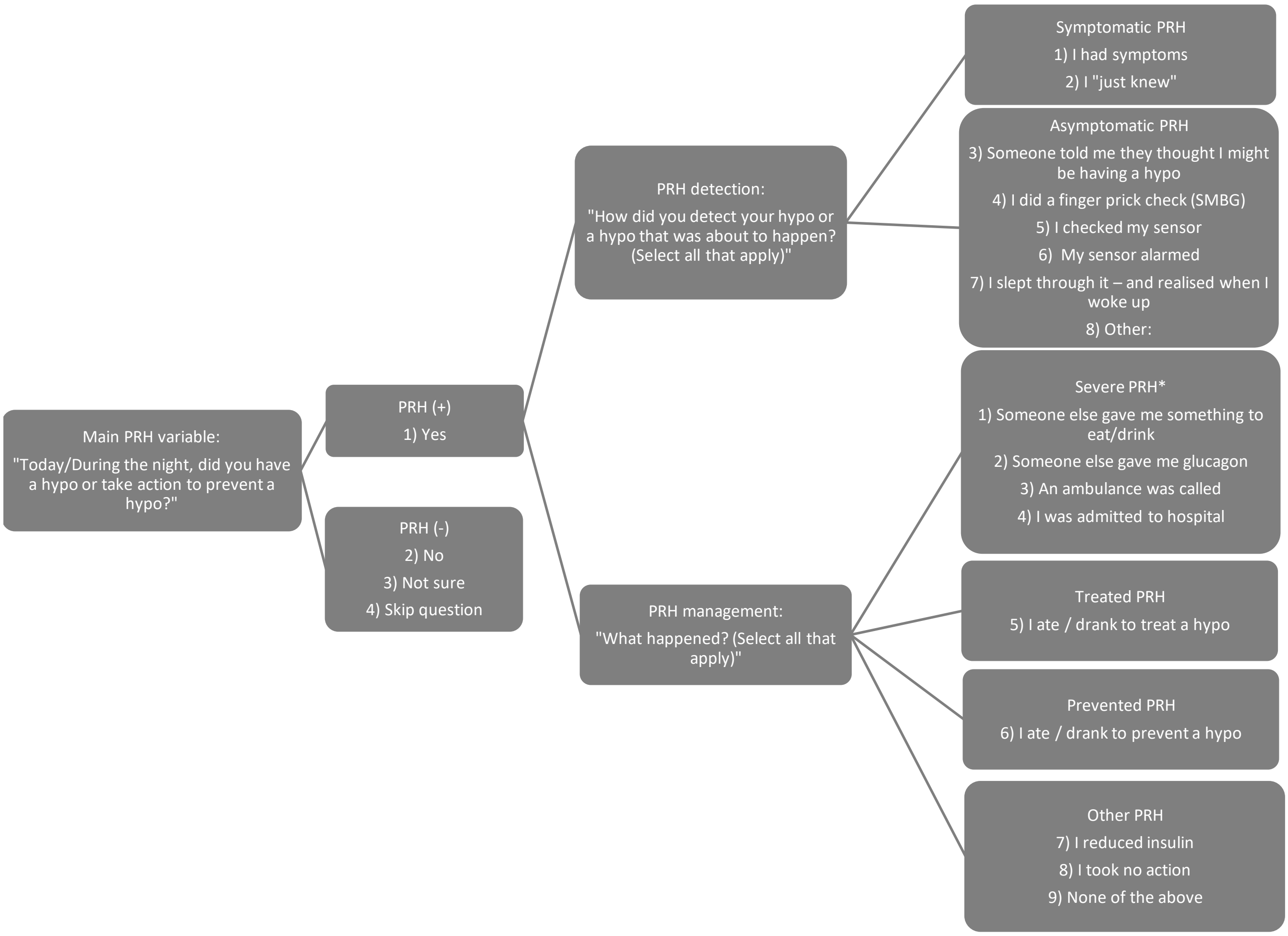

Ranking for PRH: Three separate variables were produced to represent PRH. As multiple PRH's could be reported for each night-time and daytime interval, an ordered ranking system was developed allowing each night and day to be labelled with the type of PRH that was hypothesised to have the most negative impact on daily functioning. PRH with the hypothesised most negative impact is listed towards the top of the figure; for example a PRH(+) would rank higher than a PRH(-) in the first variable, a 'Symptomatic PRH' would rank higher than a 'Asymptomatic PRH' in the second variable, and a 'Treated PRH' would rank higher than a 'Prevented PRH in the third variable.

1. Søholm, U., et al., *Psychometric properties of an innovative smartphone application to investigate the daily impact of hypoglycemia in people with type 1 or type 2 diabetes: The Hypo-METRICS app.* PLoS One, 2023. **18**(3): p. e0283148.

Participant No		Participant organisation name	Scientific person(s) in charge
1	RADBOUDUMC(Coo)	STICHTING RADBOUD UNIVERSITAIR MEDISCH CENTRUM	Prof. Cees Tack<br>Dr Bastiaan de Galan
2	KCL	King's College London	Prof. Stephanie Amiel<br>Dr Pratik Choudhary
3	MUG	Medical University of Graz	Prof. Thomas Pieber<br>Dr Julia Mader
4	UCAM	University of Cambridge	Dr Mark Evans
5	CHUM	Montpellier University Hospital	Prof. Eric Renard
6	SDU	University of Southern Denmark	Prof. Frans Pouwer<br>Prof. Jane Speight
7	UNIL	University of Lausanne	Prof. Bernard Thorens
8	USFD	University of Sheffield	Prof. Simon Heller<br>Prof. Alan Brennan
9	NOH	Nordsjællands University Hospital Hillerød	Prof. Ulrik Pedersen-Bjergaard
10	UNIVDUN	University of Dundee	Prof. Rory McCrimmon
11	Eurice	European Research and Project Office GmbH	Jakob Haardt
12	SIB	Swiss Institute of Bioinformatics	Dr Mark Ibberson

13	UNIPD	University of Padova	Prof. Giovanni Sparacino
14	UEDIN	University of Edinburgh	Prof. Helen Colhoun
15	NN	Novo Nordisk A/S	Dr Stephen Gough
16	Lilly	Eli Lilly and Company Limited	Dr Zvonko Milicevic
18	Abbott	Abbott Laboratories	Dr Mahmood Kazemi
19	MDT	Medtronic International Trading Sàrl	Dr Ohad Cohen
20	JDRF	JDRF International	Dr Sanjoy Dutta
21	IDF	International Diabetes Federation	Dominique Robert
22	T1DExchange	Unitio, Inc.	Dr Wendy Wolf
23	HCT	The Leona M. and Harry B. Helmsley Charitable Trust	Dr Sean Sullivan
